# Healthcare burden of pulmonary hypertension owing to lung disease and/or hypoxia

**DOI:** 10.1186/s12890-017-0399-1

**Published:** 2017-04-11

**Authors:** Gustavo A. Heresi, David M. Platt, Wenyi Wang, Christine H. Divers, Vijay N. Joish, Simon A. Teal, Justin S. Yu

**Affiliations:** 1 Department of Pulmonary and Critical Care Medicine, Respiratory Institute, Cleveland Clinic, 9500 Euclid Ave, Cleveland, OH 44195 USA; 2 Bayer HealthCare Pharmaceuticals, Whippany, NJ USA; 3 Currently at Regeneron, Tarrytown, NY USA; 4 Bayer Pharma AG, Berlin, Germany; 5 Currently at Allergan, Irvine, CA USA

**Keywords:** Pulmonary hypertension, Pulmonary arterial hypertension, Lung diseases, Hypoxia, Healthcare costs

## Abstract

**Background:**

Group 3 pulmonary hypertension (PH) encompasses PH owing to lung diseases and/or hypoxia. Treatment patterns, healthcare resource use, and economic burden to US payers of Group 3 PH patients were assessed.

**Methods:**

This retrospective observational study extracted data from July 1, 2010 to June 30, 2013 from two Truven Health Analytics MarketScan databases. Adult Group 3 PH patients were identified based on claims for PH (ICD-9-CM 416.0/416.8), a related lung disease, and an echocardiogram or right heart catheterization (RHC). The index date was the date of the first PH claim; data were collected for 12 months pre- and post-index. A difference-in-difference approach using generalized estimating equations was done to account for baseline differences.

**Results:**

Group 3 PH patients (*n* = 2,236) were matched 1:1 to controls on lung disease. PH patients had higher all-cause resource utilization and annual healthcare costs ($44,732 vs. $7,051) than controls. Costs were driven by inpatient admissions (35.4% of total costs), prescriptions (33.0%), and outpatient care (26.5%). Respiratory-related costs accounted for 11.4% of post-index annual costs for PH patients. PH diagnosis was not confirmed in the majority of PH patients (<7% RHC use) but nevertheless, 22% of PH patients post-index had claims for drugs approved for the treatment of pulmonary arterial hypertension (PAH).

**Conclusions:**

Group 3 PH poses a significant clinical and economic burden. Given the low use of RHC and the prevalence of PAH-indicated prescriptions that are not currently approved for Group 3 PH, this study suggests some Group 3 PH patients may not be receiving guideline-recommended treatment.

**Electronic supplementary material:**

The online version of this article (doi:10.1186/s12890-017-0399-1) contains supplementary material, which is available to authorized users.

## Background

Pulmonary hypertension (PH) is a progressive disease that may lead to decreased exercise capacity, right heart failure, and ultimately death [1]. PH is clinically defined as a resting mean pulmonary arterial pressure ≥ 25 mmHg measured by right heart catheterization (RHC) [2]. Although it can be idiopathic, PH is typically associated with a wide range of medical conditions. The World Health Organization (WHO) classifies PH owing to lung diseases and/or hypoxia as Group 3 PH [3]. Group 3 PH is a heterogeneous group and prevalence, prognosis, healthcare resource use, and economic burden vary by underlying lung disease.

Chronic lung disease is associated with a high incidence of PH. Reported prevalence rates of PH in chronic obstructive pulmonary disease (COPD) range from 30% to 70% [4]. Development of PH is also a common complication in patients with interstitial lung disease (ILD). The prevalence of PH in ILD is largely unknown because ILD encompasses a large and heterogeneous group of diseases. Prevalence rates of PH in idiopathic pulmonary fibrosis patients, the most common idiopathic ILD, range from 8% to 84% [5]. The presence and severity of PH in patients with chronic lung disease can be detrimental to patient outcomes and survival rates [1, 4, 6–8].

Regardless of WHO classification, PH can impose a substantial burden on healthcare resources and can be a costly disease. Studies have found significant healthcare resource utilization and economic burdens associated with WHO Group 1 PH (pulmonary arterial hypertension, PAH) and Group 4 PH (chronic thromboembolic pulmonary hypertension, CTEPH) [9–16]. However, there is no similar study for Group 3 PH patients as a whole in the US medical literature. The objective of this study was to assess treatment patterns, real-world healthcare resource use, and the economic burden to third-party payers of Group 3 PH patients.

## Methods

### Study design

This retrospective observational cohort study extracted data from the study period of July 1, 2010 to June 30, 2013 from two Truven Health Analytics MarketScan databases: the Commercial Claims and Encounters Database and the Medicare Supplemental Database (access granted under data licensing agreement with Truven Health Analytics). These databases contain fully paid and adjudicated healthcare claims across the US, are de-identified, and are fully compliant with the 1996 Health Insurance Portability and Accountability Act [17]. Therefore, this study did not require institutional review board or independent ethics committee approval. Approval by an institutional review board was not required. Patients were selected within the identification period of July 1, 2011 through June 30, 2012 based on ICD-9-CM (*International Classification of Disease*, *Ninth Revision*, *Clinical Modification*) codes of 416.0 (primary PH) or 416.8 (other chronic pulmonary heart disease). The date of the first PH claim was defined as the index date. The baseline and the follow-up periods consisted of 12 months pre- and post-index date, respectively.

### Patients

Group 3 PH patients were identified based on ≥2 claims (inpatient or outpatient) for PH (ICD-9-CM 416.0 or 416.8) during the identification period that were separated by at least 1 day but within 12 months of each other; ≥1claim for RHC or echocardiogram during the baseline period or before the second PH claim; and ≥1 claim for a lung disease associated with Group 3 PH during the baseline period (see Additional file 1 for the ICD-9-CM codes matched to lung disease).

Control patients were identified during the same period if they had ≥1 claim for a lung disease associated with Group 3 PH and if they lacked claims for PH (ICD-9-CM 416.0/416.8). Control patients were excluded if they had a prescription claim for prostacyclin analogues or endothelin receptor antagonists (ERAs) during the entire study period. Phosphodiesterase type-5 (PDE5) inhibitor use was not excluded as PDE5 inhibitors have other indications not related to the treatment of PH (e.g. erectile dysfunction or benign prostatic hyperplasia with or without concomitant erectile dysfunction).

All patients were required to have continuous medical and pharmaceutical coverage during the entire study period and be ≥18 years old at the index date. Patients were excluded if they had a claim code for a procedure or medical diagnosis associated with WHO PH Groups 2, 4, or 5 based on the 2013 Nice Classification during the entire study period (see Additional file 2 for the ICD-9-CM codes matched to WHO Group 2, 4, and 5 PH).

Controls were matched to cases using a two-step matching process: Group 3 PH patients and controls were pre-matched by age, sex, US census region, and plan type and then subsequently matched on lung disease(s). Controls were assigned the index date of the matched case.

### Study measures

Patient demographics examined included age, sex, census region, and type of health plan. Comorbid conditions were identified at baseline and used to calculate the Charlson comorbidity index (CCI). Outcome measures derived from claims data included prescription drug claims, outpatient physician office visits (referred to as “physician office”), all other outpatient visits (referred to as “outpatient”), inpatient hospital admissions, and emergency department (ED) visits. ED visits that led to hospitalization were counted as both an ED visit and an inpatient hospitalization. Resource use and costs were extracted 12 months pre- and post-index. Costs are presented as annual direct medical costs paid by the payer and were adjusted to 2013 US dollars using the Consumer Price Index.

### Statistical analysis

All variables were analyzed descriptively with the appropriate statistical methods: categorical variables by frequency tables (absolute and relative frequencies) and continuous variables by sample statistics (mean and standard deviation). Continuous variables were described by absolute value and as the change from baseline per analysis time point, if applicable.

Descriptive statistics (means and percentages) were calculated for demographic characteristics and clinical data. The Chi-square test for categorical variables and Student’s *t*-test for continuous variables were used to compare across groups. Healthcare resource utilization and costs were compared between the two groups at baseline and follow-up using a difference-in-difference (DID) approach with generalized estimating equations (GEE) to control for observed pre-existing baseline differences between the two patient groups. Details on the DID approach can be found in the literature [18]. The GEE analyses was also done to adjust for baseline age, sex, region, health plan type and CCI. Statistical significance was defined as *P* value < .05. Analyses were performed using SAS Software, version 9.4 (SAS Institute, Cary, NC).

## Results

### Patients

**Fig. 1:**
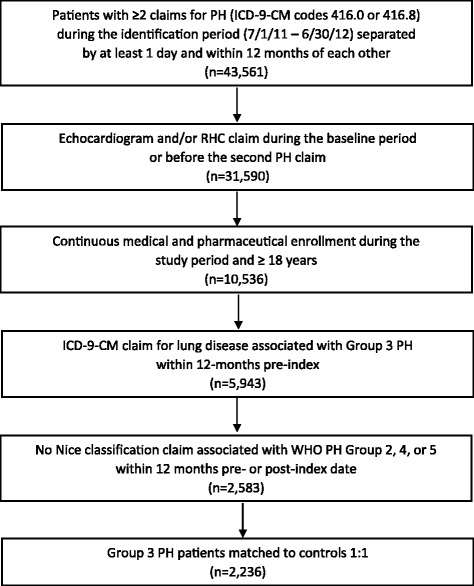
Patient Selection. Patient data were extracted from two MarketScan databases. The index date was defined as the first claim for ICD-9-CM code 416.0 (primary pulmonary hypertension) or 416.8 (other chronic pulmonary heart disease). The baseline and follow-up periods consisted of the 12 months pre- and post-index date. Patients underwent two matching procedures: case and control patients were pre-matched by age, sex, region, and plan type and subsequently matched on lung disease(s) from the pre-matched patient pool. ICD-9–CM = International Classification of Diseases, Ninth Revision, Clinical Modification; PH = pulmonary hypertension; RHC = right heart catheterization; WHO = World Health Organization

Figure 1 outlines the study criteria and the patient counts at each step; 2,236 Group 3 PH patients met the study criteria and were matched to controls in a 1:1 ratio based on lung disease(s). Patient baseline characteristics are summarized in Table 1. Compared to controls, the Group 3 PH cohort was younger (67.0 years vs. 71.0, *P* < .001), had a higher percentage of female patients (64 vs. 58%, *P* < .001), and had a higher comorbid burden (CCI score of 2.80 vs. 2.09, *P* < .001). COPD was the most common lung disease (71.1% of patients), followed by developmental lung disease (22.5%), and interstitial lung disease (19.6%).

**Table 1:** Demographic and baseline characteristics of the study population

Characteristic	Group 3 PH (*n* = 2236)	Controls (*n* = 2236)	*P* value
Age at index date, mean (SD), y	67.0 (14.4)	71.0 (11.7)^a^	<.001^b^
Age group, y			
18–30	34 (1.5)	9 (0.4)	
31–45	134 (6.0)	49 (2.2)	
46–65	841 (37.6)	596 (26.7)	
66–85	1035 (46.3)	1383 (61.9)	
>86	192 (8.6)	199 (8.9)	
Sex			
Female	1433 (64.1)	1300 (58.1)	<.001^c^
Male	803 (35.9)	936 (41.8)	
US census region			
North Central	758 (33.9)	933 (41.7)	<.001^c^
Northeast	344 (15.4)	260 (11.6)	
South	722 (32.3)	669 (29.9)	
West	401 (17.9)	372 (16.7)	
Unknown	11 (0.5)	2 (0.09)	
Health plan type			
Comprehensive	879 (39.3)	1368 (61.2)	<.001^c^
Health maintenance organization	241(10.8)	218 (9.8)	
Point-of-service	136 (6.1)	101 (4.5)	
Preferred provider organization	866 (38.7)	505 (22.6)	
Others	114 (5.1)	44 (2.0)	
CCI^d^, mean (SD)	2.80 (2.02)	2.09 (1.70)	<.001^b^
Underlying lung disease			
COPD	1590 (71.1)	1590 (71.1)	
Interstitial lung disease	438 (19.6)	438 (19.6)	
Sleep disorder breathing	340 (15.2)	340 (15.2)	
Developmental lung disease	503 (22.5)	502 (22.5)	
Alveolar hyperventilation disorder	6 (0.2)	7 (0.3)	

Values are expressed as No. (%) unless otherwise indicated. Some patients had multiple underlying lung diseases and therefore percent total is >100%. *P* < .05 was considered significant

*CCI* Charlson comorbidity index, *COPD* chronic obstructive pulmonary disease, *PH* pulmonary hypertension, *SD* standard deviation

^a^Controls were assigned the index date of the matched Group 3 PH patient

^b^Student’s *t*-test

^c^ *χ* ^2^ test

^d^Deyo adaptation of the CCI with several procedure codes that reflect the Romano adaptation

### Diagnosis and treatment

Group 3 PH patients underwent more diagnostic procedures, received more therapeutic treatment, and had more prescription pharmacy claims than control patients (Table 2). The most frequent diagnostic procedure was electrocardiography; the most frequent therapeutic treatment was ventilation perfusion. Group 3 PH patients had more claims for cardiovascular-related prescriptions (oral anticoagulants, diuretics, and calcium channel blockers) than control patients (Table 2). Group 3 PH patients also had higher rates of pharmacy claims for PAH-related drugs—prostacyclin analogues, ERAs, and/or PDE5 inhibitors—than controls in both the baseline (16.4% in Group 3 PH patients vs. 1.5% in controls) and follow-up periods (22.1 vs. 1.8%) (Table 2). PAH-related pharmaceutical use was highest in patients with comorbid ILD (31.1% use of PDE5 inhibitors, 23.3% ERAs, and 3.4% prostacyclin analogues) and COPD (16.0%, 8.6%, and 1.4%, respectively) (Table 3).

**Table 2:** Diagnostic procedures and treatment of group 3 PH patients and control patients

	Baseline		Follow-up	
	Group 3 PH (*n* = 2236)	Controls (*n* = 2236)	Group 3 PH (*n* = 2236)	Controls (*n* = 2236)
Diagnostic procedures				
Echocardiography	1679 (75.1)	518 (23.2)	1678 (75.0)	441 (19.7)
Stress echocardiography	313 (14.0)	176 (7.9)	212 (9.5)	150 (6.7)
Other radiography	107 (4.8)	77 (3.4)	79 (3.5)	59 (2.6)
Electrocardiography	1779 (79.6)	1264 (56.5)	1777 (79.5)	1160 (51.9)
Computerized tomography	1001 (44.8)	693 (31.0)	1030 (46.1)	567 (25.4)
Angiography	385 (17.2)	187 (8.4)	385 (17.2)	122 (5.5)
RHC	144 (6.4)	15 (0.7)	135 (6.0)	6 (0.3)
MRI	39 (1.7)	17(0.8)	52 (2.3)	29 (1.3)
Other exercise testing	452 (20.2)	89 (4.0)	574 (25.7)	84 (3.8)
Therapeutic treatment				
Vena cava procedure	0	0	19 (0.9)	2 (0.09)
Ventilation perfusion	76 (3.4)	12 (0.5)	58 (2.6)	2 (0.09)
Oxygen therapy	1 (0.04)	1 (0.04)	3 (0.1)	2 (0.09)
Pharmacotherapy	366 (16.4)	33 (1.5)	495 (22.1)	41 (1.8)
Oral anticoagulants	704 (31.5)	442 (19.8)	853 (38.2)	456 (20.4)
Diuretics	1273 (56.9)	714 (31.9)	1447 (64.7)	722 (32.3)
Calcium channel blockers	800 (35.8)	624 (27.9)	853 (38.2)	621 (27.8)
Digoxin	200 (9.0)	85 (3.8)	249 (11.1)	91 (4.1)
PAH-approved pharmaceuticals^a^	366 (16.4)	33 (1.5)	495 (22.1)	41 (1.8)
Prostacyclin analogues	19 (0.9)	0	34 (1.5)	0
ERAs	182 (8.1)	0	226 (10.1)	0
PDE5 inhibitors^b^	279 (12.5)	33 (1.5)	394 (17.6)	41 (1.8)

Values are expressed as No. (%)

*ERA* endothelin receptor antagonist, *MRI* magnetic resonance imaging, *PDE5* phosphodiesterase type-5, *PH* pulmonary hypertension, *RHC* right heart catheterization

^a^Includes unique patients with ≥1 pharmaceutical claim for prostacyclin analogues, endothelin receptor antagonists, and/or PDE5 inhibitors

^b^PDE5 inhibitors include those indicated for pulmonary arterial hypertension (PAH), as well as those for other indications or used off-label

**Table 3:** Key diagnostic procedures and PH-related pharmaceutical claims during follow-up by underlying lung disease

Therapy	Comorbid COPD		Comorbid ILD		Comorbid SDB		Comorbid DLD	
	Group 3 PH (*n* = 1590)	Controls (*n* = 1590)	Group 3 PH (*n* = 438)	Controls (*n* = 438)	Group 3 PH (*n* = 340)	Controls (*n* = 340)	Group 3 PH (*n* = 503)	Controls (*n* = 502)
Diagnostic Procedure								
Echocardiography	1181 (74.3)	313 (19.7)	340 (77.6)	85 (19.4)	252 (74.1)	66(19.4)	374 (74.4)	124 (24.7)
RHC	92 (5.8)	2 (0.1)	32 (7.3)	4 (0.9)	16 (4.7)	2 (0.6)	32 (6.4)	2 (0.4)
Pharmaceutical Treatment								
Oral anticoagulants	642 (40.3)	331 (20.8)	137 (31.3)	75 (17.1)	122 (35.9)	81 (23.8)	197 (39.2)	138 (27.5)
Diuretics	1084 (68.2)	521 (32.8)	236 (53.9)	126 (28.8)	224 (65.9)	134 (39.4)	359 (71.4)	185 (36.9)
Calcium channel blockers	600 (37.7)	428 (26.9)	172 (39.3)	147 (33.6)	138 (40.6)	105 (30.9)	214 (42.5)	154 (30.7)
Digoxin	194 (12.2)	67 (4.2)	46 (10.5)	17 (3.9)	22 (6.5)	13 (3.8)	64 (12.7)	23 (4.6)
Prostacyclin analogues	22 (1.4)	0	15 (3.4)	0	4 (1.2)	0	7 (1.4)	0
ERAs	137 (8.6)	0	102 (23.3)	0	25 (7.4)	0	32 (6.4)	0
PDE5 inhibitors^a^	255 (16.0)	25 (1.6)	136 (31.1)	6 (1.4)	39 (1.7)	17 (0.8)	74 (14.7)	6 (1.2)

Data are given as No. (%). Follow-up refers to the 12-month period after the index date

*COPD* chronic obstructive pulmonary disease, *DLD* developmental lung diseases, *ERA* endothelin receptor antagonist, *ILD* interstitial lung disease, *PDE5* phosphodiesterase type-5, *RHC* right heart catheterization, *SDB* sleep disorder breathing

^a^PDE5 inhibitors potentially include those indicated for pulmonary arterial hypertension (PAH), for other indications, or used off-label

### Healthcare resource use and costs

Group 3 PH patients had higher all-cause and respiratory-related resource use than control patients (Tables 4 and 5). From baseline to follow-up, all-cause resource use increased for Group 3 PH patients in all five categories examined, whereas the number of inpatient admissions and physician office visits decreased for control patients (Table 4). During follow-up, mean annual resource use per Group 3 PH patient was 86.1 prescription claims, 24.6 outpatient visits, 20.3 physician office visits, 6.2 inpatient admissions, and 0.9 ED visits. The adjusted DID was statistically significant across all five categories of resource use.

**Table 4:** Annual resource utilization and direct costs

Type	Group 3 PH			Controls			Unadjusted DID (95% CI)	Adjusted DID (95% CI)	*P* value
	Baseline	Follow-up	Difference	Baseline	Follow-up	Difference			
All-cause utilization, n									
Inpatient admissions	3.7 (8.0)	6.2 (11.4)	2.5 (10.6)	2.5 (6.1)	2.2 (5.9)	−0.2 (6.0)	2.7 (2.2–3.2)	3.1 (2.6–3.7)	<.0001
Outpatient visits	17.6 (22.3)	24.6 (27.8)	7.0 (21.7)	9.2 (12.6)	9.6 (15.4)	0.4 (11.7)	6.6 (5.6–7.6)	6.5 (5.5–7.6)	<.0001
Physician office visits	18.6 (14.2)	20.3 (15.0)	1.7 (12.1)	14.3 (12.6)	13.6 (12.7)	−0.8 (10.0)	2.5 (1.8–3.1)	2.8 (2.1–3.5)	<.0001
ED visits	0.8 (2.1)	0.9 (2.3)	0.2 (1.8)	0.4 (0.9)	0.5 (1.1)	0.1 (1.2)	0.1 (0.02–0.20)	0.1 (0.05–0.2)	0.002
Prescription claims	78.3 (50.3)	86.1 (50.7)	7.8 (31.0)	58.8 (43.7)	61.0 (45.1)	2.2 (21.2)	5.6 (3.9–6.9)	6.1 (4.5–7.7)	<.0001
All-cause costs, US$									
Total	34,040 (71,571)	44,732 (104,621)	10,691 (89,617)	8102 (14,108)	7051 (12,887)	−1051 (15,432)	11,743 (7972–15,513)	10,240 (6266–14,214)	<.0001
Inpatient	11,485 (51,815)	15,852 (84,677)	4367 (81,147)	1764 (8963)	1015 (4668)	−749 (9548)	5116 (1728–8503)	4280 (706–7855)	.019
Outpatient	8429 (28,357)	11,875 (34,344)	3446 (23,809)	2171 (5804)	1908 (8281)	−263 (8736)	3709 (2657–4760)	3309 (2198–4421)	<.0001
Physician office	1655 (3599)	1825 (4948)	170 (4105)	853 (2285)	816 (3001)	−37 (2290)	207 (12–402)	238 (31–444)	.024
ED	372 (1916)	385 (1894)	13 (1497)	79 (389)	113 (1471)	35 (1495)	−22 (−110–66)	−36 (−130–56)	.44
Prescription	12,099 (27,938)	14,795 (29,228)	2696 (18,807)	3235 (4622)	3198 (4,371)	−37 (2412)	2733 (1947–3519)	2449 (1616–3282)	<.0001

Values are expressed as mean (SD) unless otherwise specified. Baseline and follow-up refer to the 12-month periods pre- and post-index date, respectively

“Physician office” refers to outpatient physician office visits; “outpatient” refers to all other outpatient services. ED visits that led to hospitalization were counted as both an ED visit and an inpatient visit

Costs were inflated to 2013 US $ and rounded to closest dollar. Costs reflect fully paid and adjudicated medical claims paid by a third party payer. DID of healthcare costs and utilizations between the two groups from baseline to follow-up time period were analyzed by using GEE regression models adjusting for age, sex, census region, health plan type, and CCI. *P* < .05 was considered statistically significant

*CI* confidence interval, *DID* difference-in-difference, *ED* emergency department, *SD* standard deviation

**Table 5:** Annual respiratory-related resource utilization and direct costs

Type	Group 3 PH			Controls			Unadjusted DID (95% CI)	Adjusted DID (95% CI)	*P* value
	Baseline	Follow-up	Difference	Baseline	Follow-up	Difference			
Respiratory-related utilization, n									
Inpatient admissions	1.4 (4.7)	2.2 (6.0)	0.8 (5.9)	0.8 (3.0)	0.8 (3.1)	−0.1 (2.5)	0.9 (0.6-1.2)	1.1 (0.8–1.4)	<.0001
Outpatient visits	4.0 (8.0)	5.3 (10.1)	1.3 (8.0)	1.6 (4.2)	1.6 (4.5)	−0.04 (3.9)	1.4 (1.0–1.8)	1.3 (0.9–1.7)	<.0001
Physician office visits	2.2 (3.6)	2.6 (3.7)	0.4 (3.5)	1.5 (2.3)	1.3 (2.4)	−0.2 (2.3)	0.5 (0.4–0.7)	0.7 (0.5–0.9)	<.0001
ED visits	0.1 (0.5)	0.2 (0.6)	0.0 (0.6)	0.1 (0.3)	0.1 (0.4)	0.0 (0.4)	0.01 (−0.02–0.04)	0.02 (−0.01–0.05)	0.28
Respiratory-related costs, US$									
Total	3405 (16,246)	5089 (36,980)	1684 (38,082)	862 (2736)	587 (2541)	−275 (3068)	1960 (376–3544)	1856 (177–3534)	0.0303
Inpatient	2181 (15,301)	3652 (36,218)	1472 (37,958)	316 (2112)	158 (1327)	−159 (2324)	1630 (54–3207)	1511 (−160–3182)	0.0764
Outpatient	733 (2566)	981 (2909)	248 (3138)	240 (1194)	176 (1762)	−64 (1627)	312 (165–458)	319 (164–474)	<0.0001
Physician office	156 (454)	148 (370)	−8 (433)	67 (235)	39 (166)	−28 (246)	19 (−1–40.0)	26 (4–48)	0.0191
ED visit	70 (796)	59 (526)	−11 (715)	12 (112)	11 (114)	−1 (139)	−11 (−41–20)	−2 (−34–30)	0.8875

Values are expressed as mean (SD) unless otherwise specified. Baseline and follow-up refer to the 12-month periods pre- and post-index date, respectively

“Physician office” refers to outpatient physician office visits; “outpatient” refers to all other outpatient services. ED visits that led to hospitalization were counted as both an ED visit and an inpatient visit

Costs were inflated to 2013 US $ and rounded to closest dollar. Costs reflect fully paid and adjudicated medical claims paid by a third party payer. DID of healthcare costs and utilizations between the two groups from baseline to follow-up time period were analyzed by using GEE regression models adjusting for age, sex, census region, health plan type, and CCI. *P* < .05 was considered statistically significant

*CI* confidence interval, *DID* difference-in-difference, *ED* emergency department, *SD* standard deviation

Mean annual all-cause healthcare costs were over six times higher for Group 3 PH patients than controls ($44,732 vs. $7,051) post-index (Table 4). Costs increased by 31.4% from the baseline to the follow-up periods for Group 3 PH patients, but decreased by 13.0% for control patients. Post-index healthcare costs were driven by the cost of inpatient admissions (35.4% of total costs), prescription drugs (33.0%), and outpatient care (26.5%). Adjusted DID costs were statistically significant across all categories between the two groups except for the costs due to ED visits. Quartile all-cause costs are reported in Additional file 3. Post-index, mean respiratory-related costs accounted for 11.4% and 8.3% of the total healthcare costs for the Group 3 PH patients and control patients, respectively. Quartile respiratory-related costs are reported in Additional file 4.

## Discussion

To our knowledge, this is the first US study to study the real-world disease burden of Group 3 PH patients using data derived from medical claims databases. Group 3 PH patients have substantially higher rates of all-cause healthcare resource use than control patients. The highest resource use for Group 3 PH patients was in the outcome measures of prescription drug claims, outpatient visits, and physician office visits. Despite being matched by lung disease(s), Group 3 PH patients had higher resource use at baseline than control patients, possibly related to increased healthcare aimed at managing and/or diagnosing the underlying PH or related to the higher comorbidity burden of Group 3 PH patients. After controlling for key baseline covariates using a DID model approach, the differences in resource use between the two cohorts were statistically significant across all categories of healthcare use examined.

Group 3 PH poses a significant economic burden to third-party payers that is primarily driven by costs related to inpatient admissions, prescriptions, and outpatient care. The mean, annual, total direct healthcare costs of Group 3 PH patients to payers were $44,732 (approximately $3,728 per month). Other studies have estimated the economic burden of Group 1 PH (PAH) patients and of Group 4 PH (CTEPH) patients using large claims databases [9–16]. Differences in data sources, eligibility criteria, methodology, and analytical methods among studies limit a direct comparison. However, the studies by Kirson et al. [13, 14] and Said et al. [15] use a methodology similar to ours and are the most comparable. Kirson et al. reported mean direct patient-month costs (in 2007 US$) to payers of $2,023 for PAH patients and $4,782 for CTEPH patients post diagnosis. Said et al. reported mean direct monthly costs (in 2009 US$) to payers of $4,021 for PAH patients and $6,198 for CTEPH patients post diagnosis. The costs reported in this study reinforce the existing reports that regardless of etiology, PH is a costly disease to payers. The economic burden of Group 3 PH is substantial and similar to that of patients with other serious lung diseases, such as cystic fibrosis and pleural effusions [19, 20]. In 2014, two new pharmacologic treatments for patients with idiopathic pulmonary fibrosis (IPF) were introduced to the US market and this will likely increase the cost of care for this population [21]. In the current study, patients were identified from 2011 to 2012 and thus, these new costs for patients with IPF would not have been captured, but this increase in healthcare costs among this patient group may need to be considered in future studies.

Echocardiography is recommended as an initial non-invasive diagnostic tool when PH is suspected [1, 2]. However, echocardiography fails to recognize PH in many patients while also yielding false positives, and RHC is necessary to confirm a PH diagnosis [1, 2, 6]. Therefore, our inclusion criteria included claim(s) for an echocardiogram or RHC to account for the potential use of only one diagnostic method in clinical practice. Group 3 PH cohort had high use of echocardiograms (75% use in both the baseline and follow-up) but low use of RHC (6% use in both the baseline and follow-up). While database claims may be subject to under- and miscoding, the apparent lack of RHC to confirm PH in Group 3 PH patients observed in this analysis is concerning as patients were potentially receiving off-label PAH-therapy without a confirmatory RHC.

There is no approved treatment to directly treat PH when it is associated with lung diseases and/or hypoxia, and treatment is limited to treating the underlying lung disease and/or hypoxemia [1, 6]. Pharmaceuticals approved by the Food and Drug Administration for the treatment of PAH are not approved for patients with PH related to WHO Groups 2–5 except for riociguat, a soluble guanylate cyclase stimulator that is also approved for treating patients with Group 4 PH (CTEPH) who are not surgical candidates or who failed pulmonary endarterectomy [22]. Despite this, 22% of Group 3 PH patients had a claim for prostacyclin analogues, ERAs, and/or PDE5 inhibitors in the follow-up period. The relatively high utilization of expensive PAH-targeted therapies in these patients is concerning, not only because they are not approved for Group 3 PH patients, but also because of infrequent performance of RHC to confirm the diagnosis and to clarify the hemodynamic profile. The 2015 guidelines by the European Society of Cardiology and the European Respiratory Society state that the use of PAH-approved drugs in patients with Group 3 PH is not recommended (Class III, Level C) [1]. Consistently, the American College of Chest Physicians guidelines do not recommend that treatment for PAH patients be applied to patients with other types of PH [23]. The limited published evidence on the use of these drugs in Group 3 PH patients has not shown overall benefit and may cause harm in some cases [1, 23]. It needs to be noted that PDE5 inhibitors have other indications and that the data used in this study did not allow for the determination of the prescribed indication. However, the low rate of PDE5 inhibitor claims in the matched controls suggests that PDE5 inhibitors were likely prescribed for PH in Group 3 PH patients.

The observed use of PAH-approved therapies for non-PAH patients is consistent with results from a recent cross-sectional study that assessed treatment approaches at PH specialty centers [24]. At the time of that study, FDA PAH-approved therapy consisted of PDE5 inhibitors, ERAs, and prostaglandins. The use of PAH-approved drugs in Group 3 PH patients was reported in 28 out of 30 centers. The study did not determine whether PAH therapies were used off-label, as part of compassionate use, or under an alternative diagnosis. Poor right ventricular function or evidence of heart failure were reported as compelling reasons to use PAH therapy in Group 3 PH patients with comorbid COPD (17 of 28 respondents) or comorbid IPF (20 of 28 respondents).

Our study was limited by the inherent nature of medical claims databases. Since there is no specific ICD-9-CM code for Group 3 PH, we relied on diagnostic and procedure codes to identify patients with Group 3 PH. Therefore, the accuracy in identification is inherently dependent on the quality, quantity, and accuracy of the coding procedures. Claim databases do not contain all clinical information related to the patients and therefore the diagnoses and disease severity (i.e. Gold status for COPD patients) cannot be clinically confirmed, as well as the etiology of developmental lung disease and ILD. Another limitation of this study is that the two databases used contain claims data from privately insured patients and patients with Medicare supplemental coverage and those patients may not be fully representative of Group 3 PH patients.

## Conclusions

Group 3 PH presents a large burden on healthcare utilization and poses a significant economic burden to third-party payers. Although there are no currently approved drugs to treat Group 3 PH, it is possible that the approval of new pharmacotherapies could result in better management of the disease and a reduction in healthcare resource use and cost. Further research and clinical trials are necessary to explore the use of medications approved for PAH and/or CTEPH to treat Group 3 PH patients.

## Additional files

Additional file 1: Group 3 Pulmonary Hypertension Matched to Diagnostic Claims Diagnosis Codes. Table containing the ICD-9-CM diagnostic claim codes matched to Group 3 PH subgroups by lung disease. (PDF 200 kb)

Additional file 2: WHO Group 2, 4 and 5 Pulmonary Hypertension Classifications Mapped to Diagnostic/Procedure Codes. (PDF 97 kb)

Additional file 3: Annual Per-Patient All-Cause Quartile Medical Costs. (PDF 122 kb)

Additional file 4: Annual Per Patient Respiratory-Related Quartile Medical Costs. (PDF 117 kb)

## Abbreviation

CCI: Charlson comorbidity index
COPD: Chronic obstructive pulmonary disease
CTEPH: Chronic thromboembolic pulmonary hypertension
DID: Difference-in-difference
ED: Emergency department
ERA: Endothelin receptor antagonist
ICD-9-CM: International Classification of Disease, Ninth Revision, Clinical Modification
ILD: Interstitial lung disease
PAH: Pulmonary arterial hypertension
PDE5: Phosphodiesterase type-5
PH: Pulmonary hypertension
RHC: Right heart catheterization
US: United States
WHO: World Health Organization
