# Supplementary material for: Healthcare burden of pulmonary hypertension owing to lung disease and/or hypoxia
Source: BMC Pulm Med. 2017 Apr 11;17:58. doi: 10.1186/s12890-017-0399-1 (PMC5387228; doi:10.1186/s12890-017-0399-1)
Supplement: Supplementary file 1 — Group 3 Pulmonary Hypertension Matched to Diagnostic Claims Diagnosis Codes. Table containing the ICD-9-CM diagnostic claim codes matched to Group 3 PH subgroups by lung disease. (PDF 200 kb) [file 12890_2017_399_MOESM1_ESM.pdf]

**Additional File 1.** Group 3 Pulmonary Hypertension[a] Matched to Diagnostic Claims Diagnosis Codes[b]

| Group 3 Pulmonary Hypertension | ICD-9-CM Code | Code Description |
|---|---|---|
| 3.1 Chronic Obstructive Pulmonary Disease | 490-496 | COPD |
| 3.2 Interstitial Lung Disease | 446.21, 446.4 | Goodpasture's syndrome, Wegener's granulomatosis |
| | 500-505 | Coal worker's pneumoconiosis |
| | 506.4 | Chronic respiratory conditions due to fumes or vapors |
| | 508.1, 508.8 | Chronic/other pulmonary manifestations due to radiation |
| | 515 | Post-inflammatory pulmonary fibrosis |
| | 516.0 | Pulmonary alveolar protienosis |
| | 516.1, 516.2, 516.3, 516.8, 516.9 | Idiopathic pulmonary hemosiderosis |
| | 517.2, 517.8 | Lung involvement in systemic sclerosis |
| | 710 | Systemic lupus erythematosus |
| | 710.1, 710.2, 710.3, 710.4 | Systemic sclerosis |
| | 714.81 | Rheumatoid lung |
| 3.3 Other Pulmonary Diseases with Mixed Restrictive and Obstructive Pattern | | |
| 3.4 Sleep Disorder Breathing | 327.2 | Organic sleep apnea |
| | 780.51, 780.53, 780.57 | Sleep apnea |
| 3.5 Alveolar Hyperventilation Disorder | 327.24, 327.25 | Sleep related hypoxia |
| 3.6 Chronic Exposure to High Altitude | E902.0 | Residence or prolonged visit at high altitude |
| | 993.2 | Other and unspecific effects of high altitude |
| 3.7 Developmental Lung Diseases | 518.0 | Atelectasis |
| | 518.5 | Acute respiratory distress syndrome |
| | 518.81 | Respiratory failure, acute |
| | 519.2 | Mediastinitis |
| | 756.6 | Congenital diaphragmatic hernia |
| | 770.7 | Bronchopulmonary dysplasia |
| | 516.64 | Alveolar capillary dysplasia/ with vein misalignment |
| | 748.5 | Agenesis, hypoplasia, and dysplasia of lung |
| | 516.63 | Surfactant mutations of the lung |
| | 516.62 | Pulmonary interstitial glycogenosis |
| | 516.0 | Pulmonary alveolar proteinosis |
| | 516.69 | Other interstitial lung diseases of childhood |

[a] Simonneau G, Gatzoulis MA, Adatia I, et al. Updated clinical classification of pulmonary hypertension. *J Am Coll Cardiol*. 2013;62(25 Suppl):D34-D41.

[b] Popler J, Lesnick B, Dishop MK, Deterding RR. New coding in the International Classification of Diseases, Ninth Revision, for children's interstitial lung disease. *Chest*. 2012;142(3):774-780.
