# Supplementary material for: Healthcare burden of pulmonary hypertension owing to lung disease and/or hypoxia
Source: BMC Pulm Med. 2017 Apr 11;17:58. doi: 10.1186/s12890-017-0399-1 (PMC5387228; doi:10.1186/s12890-017-0399-1)
Supplement: Supplementary file 2 — WHO Group 2, 4 and 5 Pulmonary Hypertension Classifications Mapped to Diagnostic/Procedure Codes. (PDF 97 kb) [file 12890_2017_399_MOESM2_ESM.pdf]

Additional File 2. WHO Group 2, 4 and 5 Pulmonary Hypertension Classifications[a] Mapped to Diagnostic/Procedure Codes

**Pulmonary Hypertension Groups**	**Claim Codes**	**Code Description**
Group 2. Pulmonary hypertension with left heart disease		
2.1 Left ventricular systolic dysfunction	414.10	Aneurysm of heart (wall)
2.2 Left ventricular diastolic dysfunction	425.3	Endocardial fibroelastosis
	428.1	Left heart failure
	428.2	Systolic heart failure
	428.3	Diastolic heart failure
2.3 Valvular disease	394, 424.0	Disease/disorders of the mitral valve
	395, 424.1	Disease/disorders of the aortic valve
	396	Disease of mitral and aortic valve
	746.3-7, 746.81	Congenital anomalies of mitral/aortic valves
	CPT-4 codes:33400, 33401, 33403, 33405, 33406, 33410-13, 33420, 33422, 33425-7, 33430	Mitral/aortic valve surgery
2.4 Congenital/acquired left heart inflow/outflow tract obstruction and congenital cardiomyopathies	425.1	Hypertrophic obstructive cardiomyopathy
	425.8	Cardiomyopathy in other diseases classified elsewhere
	746.8	Other specified congenital anomalies of heart
Group 4. Chronic thromboembolic pulmonary hypertension (CTEPH)	415.1	Pulmonary embolism
	V12.51	History of venous thrombosis and embolism
	CPT-4: 36010, 37620, 75825, 75940<br>ICD-9 procedure: 38.7	Vena cava filter
Group 5. Pulmonary hypertension with unclear multifactorial mechanisms		
5.1 Hematologic disorders: chronic hemolytic anemia, myeloproliferative disorders, splenectomy	282	Hereditary hemolytic anemias
	283	Acquired hemolytic anemias
	238.4	Polycythemia vera
	238.79	Other lymphatic and hematopoietic tissues
	ICD-9 proc code: 41.5	Splenectomy
5.2 Systemic disorders: sarcoidosis, pulmonary histiocytosis, lymphangioleiomyomatosis	135	Sarcoidosis
	277.89, 202.5	Histiocytosis X
	228.1	Lymphangioma any site
5.3 Metabolic disorders: glycogen storage disease, Gaucher disease, thyroid disorders	271	Disorders of carbohydrate transport and metabolism
	272.7	Gaucher disease
	240-246	Disorders of thyroid gland
5.4 Others: tumoral obstruction, fibrosing mediastinitis, chronic renal failure, segmental PH	519.2	Mediastinitis
	585	Chronic renal failure
	415.19	Pulmonary emboli
	746	Other congenital anomalies of the heart

[a] Simonneau G, Gatzoulis MA, Adatia I, et al. Updated clinical classification of pulmonary hypertension. *J Am Coll Cardiol*. 2013;62(25 Suppl):D34-D41.
