# Supplementary material for: Healthcare burden of pulmonary hypertension owing to lung disease and/or hypoxia
Source: BMC Pulm Med. 2017 Apr 11;17:58. doi: 10.1186/s12890-017-0399-1 (PMC5387228; doi:10.1186/s12890-017-0399-1)
Supplement: Supplementary file 3 — Annual Per-Patient All-Cause Quartile Medical Costs. (PDF 122 kb) [file 12890_2017_399_MOESM3_ESM.pdf]

Additional File 3. Annual Per-Patient All-Cause Quartile Medical Costs

			Quartile Range				
Variable	Mean	SD	Min	25%	50%	75%	Max
Group 3 PH Patients							
Baseline costs							
Total pay	34,040	71,571	0	5,114	10,906	30,291	1,554,699
Inpatient	11,485	51,815	0	0	418	2,962	1,438,619
Outpatient	8,429	28,357	-5,934	376	1,415	5,953	476,951
Physician office	1,655	3,599	0	176	611	1,929	60,816
ED	372	1,916	-7,849	0	0	68	51,896
Prescription	12,099	27,938	0	1,266	3,742	8,777	466,865
Follow-up costs							
Total pay	44,732	104,621	-5	6,704	14,230	42,420	2,205,708
Inpatient	15,852	84,677	0	0	1,026	3,815	2,081,403
Outpatient	11,875	34,344	0	682	2,441	9,602	543,392
Physician office	1,825	4,948	0	182	733	2,046	134,964
ED	385	1,894	-5,230	0	0	74	40,440
Prescription	14,795	29,228	-464	1,495	4,414	11,521	298,874
Control Patients							
Baseline costs							
Total pay	8,102	14,108	-656	2,109	4,668	8,853	267,842
Inpatient	1,764	8,963	0	0	0	1,141	196,893
Outpatient	2,171	5,804	-1,324	65	423	1,605	69,840
Physician office	853	2,285	0	56	267	782	64,119
ED	79	389	-1	0	0	0	8,577
Prescription	3,235	4,622	0	535	2,020	4,397	108,054
Follow-up costs							
Total pay	7,051	12,887	-71	1,711	3,930	8,058	316,436
Inpatient	1,015	4,668	0	0	0	228	75,284
Outpatient	1,908	8,281	-168	37	315	1,159	279,228
Physician office	816	3,001	0	35	237	699	108,068
ED	113	1,471	0	0	0	0	66,039
Prescription	3,198	4,371	0	495	1,877	4,297	58,624

Costs were inflated to 2013 US $ and rounded to closest dollar. Costs reflect fully paid and adjudicated medical claims paid by a third party payer.
PH = pulmonary hypertension; ED = emergency department.
