# Supplementary material for: Healthcare burden of pulmonary hypertension owing to lung disease and/or hypoxia
Source: BMC Pulm Med. 2017 Apr 11;17:58. doi: 10.1186/s12890-017-0399-1 (PMC5387228; doi:10.1186/s12890-017-0399-1)
Supplement: Supplementary file 4 — Annual Per Patient Respiratory-Related Quartile Medical Costs. (PDF 117 kb) [file 12890_2017_399_MOESM4_ESM.pdf]

Additional File 4. Annual Per Patient Respiratory-Related Quartile Medical Costs

Variable	Mean	SD	Quartile Range				
			Min	25%	50%	75%	Max
Group 3 PH Patients							
Baseline costs							
Total pay	3,405	16,246	0	81	472	1,715	366,976
Inpatient	2,181	15,301	0	0	0	106	365,941
Outpatient	733	2,566	0	0	14	366	52,201
Physician office	156	453	0	0	7	129	11,807
ED	70	796	0	0	0	0	26,024
Follow-up costs							
Total pay	5,089	36,980	0	43	493	1968	1,355,621
Inpatient	3,652	36,218	0	0	0	211	1,352,279
Outpatient	981	2,909	0	0	31	529	36,876
Physician office	148	370	0	0	0	128	6,057
ED	589	526	0	0	0	0	15,580
Control Patients							
Baseline costs							
Total pay	862	2,736	0	14	132	791	47,521
Inpatient	317	2,111	0	0	0	0	47,363
Outpatient	240	1,194	-109	0	0	43	25,525
Physician office	67	235	0	0	0	42	3,895
ED	12	112	0	0	0	0	2,782
Follow-up costs							
Total pay	587	2,541	0	0	28	353	73,896
Inpatient	158	1,327	0	0	0	0	36,493
Outpatient	176	1,762	-27	0	0	2	72,247
Physician office	39	166	0	0	0	10	3,876
ED	11	114	0	0	0	0	2,702

Costs were inflated to 2013 US $ and rounded to closest dollar. Costs reflect fully paid and adjudicated medical claims paid by a third party payer.
PH = pulmonary hypertension; ED = emergency department.
